# Causes of Diseases

**Published:** 1856-11

**Authors:** W. T. Grant

**Affiliations:** Wrightsboro’, Columbia County, Georgia


### ARTICLE III.

*Causes of Disease.* By Dr. W. T. GRANT, Wrightsboro', Columbia County, Georgia.

Where do diseases come from? is a question to which, as yet, no definite answer can be given. Its investigation is rendered exceedingly difficult by the numberless elements involved. We have to take into consideration so many circumstances, that the very comprehensiveness of the question renders it repulsive to a great many who would otherwise engage in its study. But cannot this difficulty be remedied? Cannot the circumstances to be considered, the many elements involved, be so narrowed down that we may be able to grasp the whole in one view? I think so; and it is the object of this communication to attempt the accomplishment of this object. To do this, we must elevate ourselves above the consideration of individual causes, and take a view of the whole field of diseases and their causes.

My first proposition that I would lay down in connection with this investigation is, that *no disease orignates within the system*; every disease or morbid condition, whatever its nature, finds its cause outside of the organism. And as a sequela to this proposition, I will say that it is impossible for any one to become diseased whose every organ is exercising its function in perfect health, and who is not exposed to any extraneous morbid agencies. Take any one whose system is entirely free from disease, in whom the tripod of life—the nervous system, the circulating system and the respiratory—is acting normally, and I will warrant such a person good and perfect health as long as he will keep himself free from every external morbid agent. This is a bold position to assume, but I think it the only reasonable one. If the nervous system—I mean the whole nervous system—is in perfect health, producing just the normal quantity, as well as quality, of the nervous fluid; if this fluid be properly distributed to the various parts of the system; just the right quantity to each organ and part, and if each organ or part is perfectly sound, why should not each and every part perform its function normally? And why should there not be perfect health? And, also, how can any of the parts become deranged? Let us illustrate the proposition with some organ. Take the liver for example. If the liver is acting healthily, how may it become deranged? In several ways: by receiving too much or not enough nervous fluid; by receiving too much or too little blood—or the blood may be unhealthy. Now, suppose it should receive too little nervous fluid, it will be deranged in its function of course; and we must go to the nervous centres to ascertain why enough nervous fluid has not been sent to the liver. So there we consult the nervous centres, and we will find that they cannot, perhaps, produce the *quantum sufficit* of nervous fluid. Then, the question arises, why cannot they do it? The answer is, that something is in the way. Do they stop of themselves performing their function, or does something else stop them? If they possessed volition, independent volition, they might very readily cease the performance of their function. But they have no volition, and cannot therefore work at leisure. So, then, if they have ceased to produce the proper quantity of nervous fluid, it is because they have been acted upon by some injurious agent. This agent will be found in the blood; but it is foreign to the blood; that is, it does not naturally belong in the blood, else the nervous centres would never perform their function normally. Where, then, does the blood get this deleterious agent? It may have entered the system through some one of the avenues that lead into the body, the lungs, the skin, or the stomach; or it may have been produced in the system and could not get out. If the first position be taken, then our original position is demonstrated. But if the last be taken, then I ask if the deleterious agent has been produced in the system and could not get out, *why* couldn't it get out? Nature has provided an exit for every element or compound which is produced in the system, and which may prove injurious by being retained. Then the above question is answered. If the injurious agent could not get out, it is the fault of some one of the excreting organs. What deranged that organ, then, is asked? Something deranged it of course. The agent that produced the disturbance in its function is found in the blood also, and if traced around through its windings through the system, will be found to have entered from the outside. Now, then, to recapitulate in a reverse direction to that which we have just been pursuing, suppose some deleterious agent enters the system, and, acting upon the kidneys, diminishes the excretion of urea; the urea remaining in the system will affect the nerve centres so that they cannot produce their accustomed quantity of the nervous fluid, in consequence of which the liver, as well as other organs, will not receive their due supply, and must necessarily become deranged in their function.

I conceive that I need not illustrate this point further, for, with but slight change, the above illustrations will apply to every case of disease. The reader can trace the bearings. Whether the original proposition that all diseases are produced by causes external to the system or not, is established, is immaterial in this place. I willh owevera ssume that it is proved.

Now, then, if all diseases are produced by causes external to the system, through what channels do they act upon it so as to produce disease? And what is the nature of these causes or agents? There are three modes of ingress: through the stomach, through the lungs, and through the skin. Agents acting through the stomach are mostly palpable, and I shall not consider them, as it is not my object to investigate the known causes of diseases, but the unknown. We have left, then, the remaining two—the lungs and the skin. That the skin affords easy ingress to deleterious agents, there can be no doubt. A sailor once told the writer that he was aboard a ship in the West Indies, at one time during an extremely fatal epidemic of yellow fever, and that his ship was in its very midst. An "old tar" told the seamen of his vessel to wear flannel shirts and there would be no danger, which was done by all the sailors with one exception. All escaped except the one who wore no flannel. I cannot say that there was, in this case, any virtue in the flannel, but the coincidence was singular, to say the least; and if any reliance may be placed in the statement, the flannel was prophylactic only in excluding the yellow fever *materies morbi* from the skin. Other cases on record, as well, also, as a great many observations made by different persons at different times, all go to prove that the pores of the skin afford an easy ingress to morbid agents from without. That the lungs are an avenue by which diseases find an entrance into the system, no one will now deny.

Having now indicated the channels by which the *materies*

*morbi* enter the system, I may now consider those agents themselves. Chemistry informs us that all matter exists in one of three forms: solid, liquid or gaseous. We know then that the poison of diseases exists in one of these three forms. Now, then, inasmuch as nine-tenths of the diseases enter through either the lungs or skin, and since a solid or a liquid could not pass through those organs, (excepting under extraordinary circumstances,) we are led to look among the gases for a solution of the question. Where do diseases come from? I think that we may accuse them of being the generators of nine-tenths of "the ills to which flesh is heir." This narrows the question down to the gases. Can it not be carried still further? I think so, most unquestionably; but it is in the hands of the chemist. Is not yellow fever superinduced by hydrogen or some of its compounds, or hydrogen in an allotropic condition? A very significant item on this point is the following: During the yellow fever epidemic in New Orleans in 1853, Dr. McFarlane of that city, attached a piece of perfectly fresh meat to a kite and let it up into the air, and in a very short time the meat was putrid. The agent that produces our fevers is undoubtedly gaseous in its nature. The pestiferous vapors that float over the Pontine marshes are nothing more than a solution of the deleterious agent in water. The reader has undoubtedly observed over and over again the dense fogs of an early morning: they are pregnant with danger, especially in the Autumn season; they are loaded down with health-destroying gases. It is for the very same reason that exposure to the night air is so ruinous to health. The reason is clear: at midday, when the sun shines bright and warm upon the various bodies of water around, an evaporation takes place from their surface; and whenever this evaporation occurs from a marsh, the vapor rises highly charged with the soluble gases which are produced by the decay of organic matter. The vapor remains suspended in the atmosphere, and when the sun has set, and his heat is withdrawn, the earth and other bodies begin to cool; and the vapor in the atmosphere being thus exposed to a cooling process, is condensed in the form of dew, and gives up its cargo of dangerous gases, and they float in the atmosphere until disposed of in some way by the laws of nature. There are ten thousand causes at work around us every minute, the very existence of which we do not even suspect. Allotropism may be at work, arranging and rearranging the elements of nature around us, changing their properies and nature, devising new compounds that the chemists never dreamed of. It may be constantly altering the nature of the simple elements with which the chemist deals, and then sending them into our bodies to act catalytically upon the elements that go to make up our blood as well as other parts of our bodies. Here is the key to physiology, but it will be a long time before we may enter within the temple where we will find the philosopher's stone with which to conquer disease.

